# Complete genome sequence of three methicillin-resistant *Staphylococcus aureus* ST630 strains isolated from cattle in Algeria

**DOI:** 10.1128/mra.00995-24

**Published:** 2025-02-25

**Authors:** Chahrazed Belhout, Javier E. Fernandez, Vincent Perreten

**Affiliations:** 1 Division of Molecular Bacterial Epidemiology and Infectious Diseases, Vetsuisse Faculty, Institute of Veterinary Bacteriology, University of Bern, Bern, Switzerland; Loyola University Chicago, Chicago, Illinois, USA

**Keywords:** MRSA ST630, animals, complete genome

## Abstract

Methicillin-resistant *Staphylococcus aureus* (MRSA) sequence type 630 (ST630) is an emerging clone causing infections in humans. We report the complete genomes of three MRSA ST630 from cattle. The 2.8 Mbp genomes contained several antimicrobial resistance and virulence genes and were related to those of previously detected human-associated MRSA ST630.

## ANNOUNCEMENT

*Staphylococcus aureus* is an opportunistic pathogen causing infections in both the community and hospitals (1). Methicillin-resistant *S. aureus* (MRSA) sequence type (ST) 630 recently emerged as a single allele variant (*arcC*) of ST8, a hypervirulent community-acquired MRSA disseminating worldwide (2, 3). Unlike ST8, MRSA ST630 is still rare and so far only associated with human infections in China and Denmark (4, 5), and not yet in animals.

Three MRSA were isolated on Columbia agar (Oxoid) containing 5% defibrinated sheep blood and 3.5 mg/L cefoxitin after incubation at 37°C for 24 h from the nasal swabs of healthy cattle from different farms in Algeria in 2022 (Table 1). Isolates were purified on trypticase soy agar containing 5% sheep blood (TSA-SB) (Becton Dickinson), identified as *S. aureus* using matrix-assisted laser desorption ionization-time of flight mass spectrometry (Bruker), and cryopreserved in our collection.

**TABLE 1:** Sequencing statistics and genomic characteristics of MRSA ST630-t4549 strains CBC240, CB235, and CB226 isolated from the nasal cavity of cattle^*b*^

Parameters	Results for MRSA ST630 strains		
	CBC240	CBC235	CBC226
Origin	Cattle, farm 1	Cattle, farm 2	Cattle, farm 3
Sample	Nasal swabs	Nasal swabs	Nasal swabs
Region	Birtouta, Algiers	El-Harrach, Algiers	Djassr Kasentina, Algiers
GenBank accession numbers	CP169267	CP169266	CP169265
Illumina statistics			
SRA accession numbers	SRR30352266	SRR30352265	SRR30352264
Read length	36–151	36–151	36–151
Number of paired reads	24,91,950	20,51,627	19,91,476
Coverage (×)	260.6	214.5	209.1
Oxford Nanopore statistics			
SRA accession numbers	SRR30352263	SRR30352262	SRR30352261
*N*_50_ (bp)	19,363	17,814	15,173
Mean read length (bp)	12,457.30	11,772.60	10,261.10
Number of reads	40,608	38,553	30,986
Coverage (×)	176.3	158.2	110.8
Size and GC content			
Total length (bp)	28,68,940	28,68,882	28,68,882
Coverage (×)	437	373	320
GC content (%)	32.73	32.73	32.73
Number of			
Predicted genes	2,872	2,871	2,871
CDSs	2,790	2,789	2,789
rRNAs	19	19	19
tRNAs	62	62	62
tmRNAs	1	1	1
SNPs**^*a*^**			
CBC240/CBC235/CBC226	0/5/5	5/0/4	5/4/0
SNPs’ position			
CBC240	–^*c*^	C379,368T; G416,831C; A1,014,380G; T1,738,128A; T2,645,132G	C379,368T; G416,831C; A1,014,378G; C1,148,938A; C1,961,774T
			
CBC235	C379,368T; G416,831C; A1,014,380G; T1,738,128A; T2,645,132G	–	C1,038,111T, T1,738,127A, G1,821,953A, G2,518,872A
CBC226	C379,368T; G416,831C; A1,014,378G; C1,148,938A; C1,961,774T	C1,038,111T, T1,738,127A, G1,821,953A, G2,518,872A	–
ANI (%)			
CBC240/CBC235/CBC226	100/99.98/99.98	99.98/100/99.99	99.98/99.99/100

^*a*^ The coverage in the SNP regions was evenly distributed among strains with a minimum coverage of 33× and an average coverage of 93×.

^*b*^ CDSs, coding DNA sequences; rRNAs, ribosomal RNAs; tRNAs, transfer RNAs; tmRNAs, transfer-messenger RNAs; SNPs, single nucleotide polymorphisms; and ANI, average nucleotide identity.

^*c*^ "–" indicates not applicable since it consists of the same strain.

**Fig 1:**
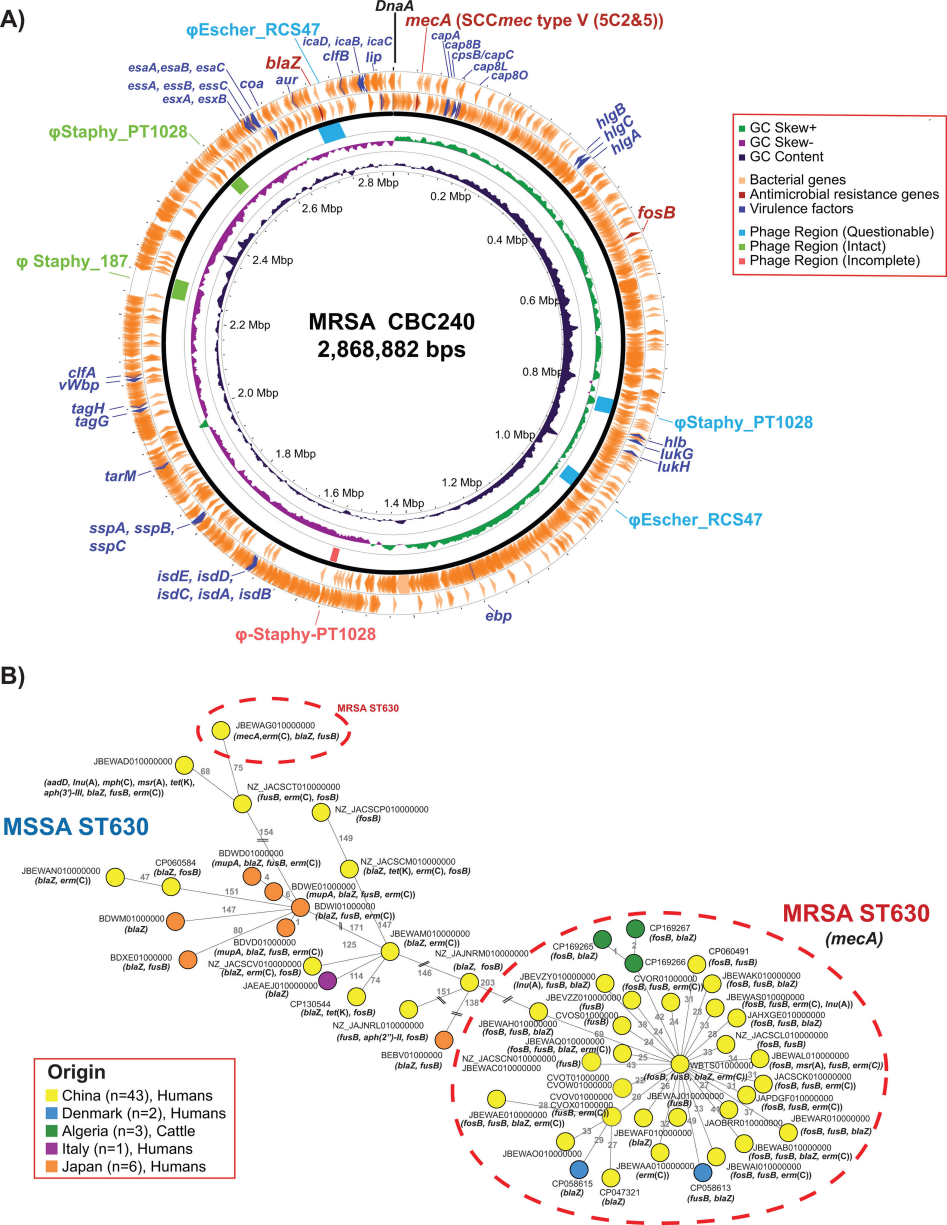
Circular genome map of MRSA ST630 strain CB240 isolated from the nasal cavity of cattle in Algeria (**A**), and cgMLST-based minimum spanning tree analysis of *S. aureus* ST630 from cattle with those from the GenBank (**B**). The map includes GC content distribution, location of antibiotic resistance genes (*mecA, blaZ* for β-lactams; *fosB* for fosfomycin), virulence genes associated with hemolysins (*hlb, hlgA, hlgB,* and *hlgC*), capsule (*capA, cap8B, cpsB/capC, cap8L,* and *cap8O*), coagulase (*coa*), adhesion factors (*clfA, clfB, isdE, isdD, isdC, isdA,* and *isdB*), secretion systems (*vWbp, esaA, esaB, esaC, essA, essB, essC, esxA,* and *esxB*), exoenzymes (*aur, sspA, sspB,* and *sspC*), intact prophages (**φ**Staphy_PT1028, **φ**Staphy_187) and putative prophage regions (related to **φ**Escher_RCS47 and **φ**Staphy-PT1028). The nodes in the minimum spanning tree are color coded according to the country of origin. Groups containing MRSA ST630 strains are marked with red circles. All MRSA ST630 strains belonged to *spa* type t4549, except for JAOBRR010000000 (*spa* type t14066), JBEWAQ010000000 (t21038), JBEWAG010000000 (t377), JBEWAJ010000000, JBEWAE010000000, JBEWAT010000000 (t2196), and JBEVZY010000000, JBEWAO010000000, and JBEWAP010000000 (unknown *spa* types).

Whole genome sequences (WGS) were obtained using Illumina and Oxford Nanopore Technologies (ONT) from genomic DNA (gDNA) extracted from colonies grown at different times but from the same frozen batch on TSA-SB at 37°C for 24 h. Illumina libraries were prepared from gDNA extracted following the Nextera DNA Flex Microbial Colony Extraction protocol using the Nextera DNA flex library preparation kit and sequenced (2 × 150 bp paired-end reads) on a NovaSeq 6000 system (S4 flow cell, 300 cycles) (NGS Platform, University of Bern, Switzerland). Illumina reads were quality controlled (QC) using FastQC version 0.12.1 (6) and filtered using Trimmomatic version 0.39 (7). ONT libraries were prepared from gDNA extracted with Qiagen DNeasy Blood and Tissue Kit using the ligation kit SQK-NB114-24 and sequenced using a FLO-FLG114 flow cell R10 on the MinION Mk1B device. Base calling and demultiplexing were performed with Dorado version 0.5.3 (8), and QC using NanoPlot version 1.40.2 (9). Short and long reads were hybrid assembled and rotated to DnaA using Unicycler version 0.5.0 (10). Assemblies were QC using QUAST version 5.2.0 (11) and annotated using PGAP version 6.8 (12). STs were determined using PubMLST (13), and core genome multilocus sequence typing (cgMLST) and *spa* typing were performed using Ridom SeqSphere+ version 10.0.4 (14). Single nucleotide polymorphisms (SNPs) were called using Snippy version 3.2 (https://github.com/tseemann/snippy). Average nucleotide identity (ANI) was calculated using ANI Calculator (15). Antimicrobial resistance and virulence determinants were screened using ABRicate version 1.0.1 against ResFinder version 4.6 (16) and the Virulence Factor Database (17). SCC*mec* was typed using SCC*mec*Finder version 1.2 (https://cge.food.dtu.dk/services/SCCmecFinder/). A circular map of the chromosome, including prophage sequence prediction, was generated using PHASTEST (https://phastest.ca/) (18) (Fig. 1A). Default parameters were used for all software. WGS characteristics are listed in Table 1.

The three MRSA belonged to ST630 *spa* type t4549 and differed by four to five SNPs (Table 1). The cgMLST analysis using all GenBank-available *S. aureus* ST630 (*n* = 57) revealed that bovine MRSA ST630 clustered with others from humans differing by 0–79 alleles (Fig. 1B). All strains harbored resistance genes to fosfomycin and β-lactams with *mecA* located on a 21-kb SCC*mec* type V (5C2&5), virulence genes, and prophage-related regions (Fig. 1A). The presence of MRSA ST630 in animals highlights its potential for dissemination among humans and animals.

## Data Availability

The complete genome sequences of MRSA ST630 strains CBC240, CBC235, and CBC226 have been submitted to GenBank with the accession numbers CP169267, CP169266, and CP169265, respectively. The associated BioSample accession numbers are SAMN43318619, SAMN43318620, and SAMN43318621, respectively. The raw reads were deposited in the Sequence Read Archive (SRA) under BioProject PRJNA1151632 with SRA accession numbers SRR30352266, SRR30352265, and SRR30352264 for Illumina reads and SRR30352263, SRR30352262, and SRR30352261 for ONT reads.

## References

[1] Murray CJL, Ikuta KS, Sharara F, Swetschinski L, Robles Aguilar G, Gray A, Han C, Bisignano C, Rao P, Wool E, et al.. 2022. Global burden of bacterial antimicrobial resistance in 2019: a systematic analysis. The Lancet 399:629–655. doi:10.1016/S0140-6736(21)02724-0 PMC8841637 35065702

[2] Strauß L, Stegger M, Akpaka PE, Alabi A, Breurec S, Coombs G, Egyir B, Larsen AR, Laurent F, Monecke S, Peters G, Skov R, Strommenger B, Vandenesch F, Schaumburg F, Mellmann A. 2017. Origin, evolution, and global transmission of community-acquired Staphylococcus aureus ST8. Proc Natl Acad Sci U S A 114:E10596–E10604. doi:10.1073/pnas.1702472114 29158405 PMC5724248

[3] Lepuschitz S, Huhulescu S, Hyden P, Springer B, Rattei T, Allerberger F, Mach RL, Ruppitsch W. 2018. Characterization of a community-acquired-MRSA USA300 isolate from a river sample in Austria and whole genome sequence based comparison to a diverse collection of USA300 isolates. Sci Rep 8:9467. doi:10.1038/s41598-018-27781-8 29930324 PMC6013426

[4] Shi J, Xiao Y, Shen L, Wan C, Wang B, Zhou P, Zhang J, Han W, Yu F. 2024. Phenotypic and genomic analysis of the hypervirulent methicillin-resistant Staphylococcus aureus ST630 clone in China. mSystems 9:e0066424. doi:10.1128/msystems.00664-24 39158330 PMC11406941

[5] Sieber RN, Overballe-Petersen S, Kaya H, Larsen AR, Petersen A. 2020. Complete genome sequences of methicillin-resistant Staphylococcus aureus strains 110900 and 128254, two representatives of the CRISPR-cas-carrying sequence type 630/spa type t4549 lineage. Microbiol Resour Announc 9:00891–20. doi:10.1128/MRA.00891-20 PMC7545285 33033131

[6] Andrews S. 2010. FastQc: a quality control tool for high throughput sequence data. Available from: http://www.bioinformatics.babraham.ac.uk/projects/fastqc

[7] Bolger AM, Lohse M, Usadel B. 2014. Trimmomatic: a flexible trimmer for Illumina sequence data. Bioinformatics 30:2114–2120. doi:10.1093/bioinformatics/btu170 24695404 PMC4103590

[8] Oxford Nanopore Technologies. n.d. Dorado: Oxford Nanopore’s Basecaller. GitHub repository. Available from: https://github.com/nanoporetech/dorado

[9] De Coster W, Rademakers R. 2023. NanoPack2: population-scale evaluation of long-read sequencing data. Bioinformatics 39:btad311. doi:10.1093/bioinformatics/btad311 37171891 PMC10196664

[10] Wick RR, Judd LM, Gorrie CL, Holt KE. 2017. Unicycler: resolving bacterial genome assemblies from short and long sequencing reads. PLOS Comput Biol 13:e1005595. doi:10.1371/journal.pcbi.1005595 28594827 PMC5481147

[11] Mikheenko A, Prjibelski A, Saveliev V, Antipov D, Gurevich A. 2018. Versatile genome assembly evaluation with QUAST-LG. Bioinformatics 34:i142–i150. doi:10.1093/bioinformatics/bty266 29949969 PMC6022658

[12] Tatusova T, DiCuccio M, Badretdin A, Chetvernin V, Nawrocki EP, Zaslavsky L, Lomsadze A, Pruitt KD, Borodovsky M, Ostell J. 2016. NCBI prokaryotic genome annotation pipeline. Nucleic Acids Res 44:6614–6624. doi:10.1093/nar/gkw569 27342282 PMC5001611

[13] Jolley KA, Bray JE, Maiden MCJ. 2018. Open-access bacterial population genomics: BIGSdb software, the PubMLST.org website and their applications. Wellcome Open Res 3:124. doi:10.12688/wellcomeopenres.14826.1 30345391 PMC6192448

[14] Jünemann S, Sedlazeck FJ, Prior K, Albersmeier A, John U, Kalinowski J, Mellmann A, Goesmann A, von Haeseler A, Stoye J, Harmsen D. 2013. Updating benchtop sequencing performance comparison. Nat Biotechnol 31:294–296. doi:10.1038/nbt.2522 23563421

[15] Yoon S-H, Ha S-M, Lim J, Kwon S, Chun J. 2017. A large-scale evaluation of algorithms to calculate average nucleotide identity. Antonie Van Leeuwenhoek 110:1281–1286. doi:10.1007/s10482-017-0844-4 28204908

[16] Zankari E, Hasman H, Cosentino S, Vestergaard M, Rasmussen S, Lund O, Aarestrup FM, Larsen MV. 2012. Identification of acquired antimicrobial resistance genes. J Antimicrob Chemother 67:2640–2644. doi:10.1093/jac/dks261 22782487 PMC3468078

[17] Chen L, Zheng D, Liu B, Yang J, Jin Q. 2016. VFDB 2016: hierarchical and refined dataset for big data analysis—10 years on. Nucleic Acids Res 44:D694–7. doi:10.1093/nar/gkv1239 26578559 PMC4702877

[18] Wishart DS, Han S, Saha S, Oler E, Peters H, Grant JR, Stothard P, Gautam V. 2023. PHASTEST: faster than PHASTER, better than PHAST. Nucleic Acids Res 51:W443–W450. doi:10.1093/nar/gkad382 37194694 PMC10320120

